# Endometriosis and IVF treatment outcomes: unpacking the process

**DOI:** 10.1186/s12958-023-01157-8

**Published:** 2023-11-07

**Authors:** Edgardo Somigliana, Letizia Li Piani, Alessio Paffoni, Noemi Salmeri, Michele Orsi, Laura Benaglia, Paolo Vercellini, Paola Vigano’

**Affiliations:** 1 Academic Center for Research on Adenomyosis and Endometriosis, Department of Clinical Sciences and Community Health, Università degli Studi di Milano, Milan, Italy; 2 Dipartimento Area Materno Infantile, Fondazione IRCCS Ca’ Granda Ospedale Maggiore Policlinico, Milan, Italy; 3 Department of Clinical Sciences and Community Health, Università degli Studi di Milano, Milan, Italy; 4 ASST Lariana, Infertility Unit, Como, Italy; 5 Gynecology and Obstetrics Unit, IRCCS San Raffaele Scientific Institute and Vita-Salute San Raffaele University, Milan, Italy

**Keywords:** Endometriosis, Endometrioma, IVF, ART, Oocyte, Embryo, Implantation

## Abstract

Advanced endometriosis is associated with a reduction of IVF success. Surgical damage to the ovarian reserve following the excision of endometriomas has been claimed as a critical factor in the explanation of this detrimental effect. However, it is generally inferred that other mechanisms might also hamper IVF success in affected women. They include diminished responsiveness to ovarian stimulation, altered steroidogenesis, a decline in oocyte quality, reduced fertilization and embryo development, and impaired implantation. To navigate these limitations, we scrutinized available literature for studies specifically designed to address distinct phases of the IVF process. Utmost consideration was given to intra-patient ovarian response comparisons in women with unilateral endometriomas and to studies applying a meticulous matching to control confounders. The following observations have been drawn: 1) endometriosis has a negligible impact on ovarian response. A slight reduction in stimulation response can only be observed for endometriomas larger than 4 cm. Follicular steroidogenesis is unaffected; 2) oocyte quality is not hampered. Fertilization rates are similar, and intracytoplasmic sperm injection (ICSI) is not justified. Embryonic development is uncompromised, with no increase in aneuploidy rate; 3) endometrial receptivity is either unaffected or only slightly impacted. In conclusion, our study suggests that, aside from the well-known negative effect on ovarian reserve from excisional endometrioma surgeries, endometriosis does not significantly affect IVF outcomes.

## Introduction

A substantial body of literature has examined the connection between endometriosis and infertility [1, 2]. It is likely that a multifactorial process underlies the strong clinical association between the two conditions. Pelvic adhesions and chronic pelvic inflammation may interfere with processes such as ovulation, oocyte uptake, sperm transport and function, gamete fertilization, and embryo migration and implantation. The association of endometriosis with other fertility-impairing conditions such as adenomyosis might also play a role [2, 3]. Assisted reproductive technology (ART) procedures can overcome some of these adverse phenomena by controlling for a wide range of infertility issues, including ovulation disorders, fertilization failure, and tubal damage. Nonetheless, women with endometriosis may still face challenges during ART cycles. In a meta-analysis published in 2013, Harb et al. reported an impairment of IVF success in women with advanced endometriosis (with a relative risk of clinical pregnancy of  0.79, 95% CI 0.69–0.91), but failed to show an impact on live births [4]. A more recent meta-analysis showed a significant decrease in live births in women with stage III-IV endometriosis (with an odds ratio of 0.78, 95% CI 0.65–0.95) [5]. In addition, available meta-analyses suggested a lower number of oocytes retrieved in affected women [6], as well as lower peripheral estrogens levels at the time of trigger [7].

Surgery for endometriomas has been claimed as a major factor in interpreting these outcomes. A detrimental effect of endometrioma excision on ovarian reserve has already been extensively reported [8–12]. Serum Anti-Mullerian Hormone (AMH) shrinks after surgery [10], and the ovarian response to stimulation is halved [6, 13]. In one out of every eight operated gonads, the ovarian reserve is worn out [14]. Of utmost relevance, the lower is the remnant follicular pool, the fewer is the number of oocytes retrievable during IVF, resulting in lower cumulative live birth chances per retrieval [15]. Of note, some detrimental effect may precede surgery. Muzii et al. reported slightly lower AMH levels in women with endometriomas (mean difference in patients with unoperated endometriomas compared to patients with no endometriomas -0.84, with 95% confidence interval [CI] -1.16 to -0.52) [16].

Thus, damage to the ovarian reserve as a result of surgery might not be the unique determinant of IVF success rates in women with endometriosis. It has been argued that the disease can hinder other crucial IVF steps, such as ovarian response, oocyte quality, and embryo implantation [1, 17]. On the other hand, these claims lack robust evidence. In our opinion, this topic is in need of in-depth investigation. Discerning potential mechanisms that might impair IVF success beyond the surgical damage to the ovarian reserve is fundamental. Such insights may suggest therapeutic approaches or add-ons to boost procedure success and can shed more light on mechanisms impeding natural conception.

### The morass of confounders and possible solutions

Relying on meta-analyses in order to discern the adverse effects of endometriosis can be misleading. At least, it may not be informative for the purpose of the present study—disentangling which steps of the IVF procedure are negatively affected. Observational studies comparing IVF outcomes in women with and without endometriosis usually have important limitations that meta-analyses cannot obviously overcome. Of greatest relevance, there is often a lack of appropriate adjustment for the damage to the ovarian reserve. This factor has a pivotal impact on success rates. Additional shortcomings include: (i) the negligence of the strong association between adenomyosis and endometriosis, impairing the possibility to disentangle the independent detrimental effects of the two conditions [3]; ii) adjustment for additional confounders (besides previous surgeries and adenomyosis) is often not done, or performed relying on arbitrary statistical models; (iii) diagnostic criteria across studies are highly heterogenous; (iv) most studies have not separately evaluated women with the lesions in situ and those who have previously undergone lesion excision; (v) studies often do not take into account that endometriosis-related lesions are highly heterogeneous; even if studies attempt to focus on specific types of endometriosis, they cannot avoid including women with multiple forms of the disease in the same group; vi) controls may have higher or lower chance of success, as not all causes of infertility yield similar IVF success rates.

Over the last two decades, two appealing methodological strategies have been used by our and other groups to address confounders and to isolate the effects of the disease on different IVF phases: the within-patient comparison of ovarian response in women with unilateral endometrioma and the matching design approach (Table 1).

**Table 1:** Study designs used to limit confounders when investigating the impact of endometriosis on IVF

Study design	Description	Pros	Cons
Intrapatient comparisons of the two ovaries	The ovarian response is compared between the affected ovary and the contralateral intact gonad of the same patient	Ovaries in the same conditions	Non informative on pregnancy rate
		Powerful statistics (paired analyses)	Ovarian response in terms of number of follicles is reliable also for retrospective studies. Data on the quality of the oocytes needs prospective recruitment
Matching	Women with endometriosis are matched by age and study period to 1 or more controls (matching 1:1, 1:2 or even more). Matching can be done for other additional variables depending on the investigated item	It allows to study also the impact on clinical pregnancy rate or live birth rate	Residual biases can remain (the intrapatient comparisons are less vulnerable)
		It can be used to study endometriosis in general, not only ovarian endometriomas	The detrimental effects of unilateral lesions can be missed or diluted

#### Intra-patient comparison (affected versus unaffected contralateral ovary)

For ovarian endometriomas, an informative study design is the comparison with the unaffected contralateral ovary in women with unilateral disease [18]. The strength of this design is that it uses the woman as her own control, ensuring that the affected and the contralateral intact ovaries experience identical conditions. This approach also boosts the statistical power of the analyses through paired comparisons. Both retrospective and prospective studies can be performed, with the latter being informative also on the folliculogenesis quality (as oocytes retrieved can be kept and observed separately) rather than just on the ovarian response quantity. However, such design does not provide data on the chances of pregnancy. An example of this design is the prospective study from Ragni et al. (2005) evaluating the surgical-related ovarian damage [18]. Thirty-eight women operated for a unilateral endometrioma were recruited and ovarian response of the operated ovary was compared with the contralateral intact gonad. A reduced number of dominant follicles, oocytes, embryos, and high-quality embryos was observed in the operated gonad. Reduction percentages averaged 60% (95% CI: 38–81%), 53% (95% CI: 30–75%), 55% (95% CI: 28–81%), and 52% (95% CI: 17–87%), respectively. On the other hand, both fertilization and good-quality embryos rates were similar between the two ovaries. The authors concluded that surgery caused a quantitative, but not a qualitative, ovarian damage [18].

#### Meticulous matching

A scrupulous matching between women with and without endometriosis can help addressing significant confounders. The current spread of the use of propensity score matching supports the validity of this approach. This study design is mainly retrospective but, unlike the intra-patient comparison, it can also provide valuable insights on pregnancy rates. Furthermore, it allows to study endometriosis in general, not just endometriomas. The matching design, compared to multivariate analyses, benefits from not relying on a predetermined statistical model. Essential matching variables include age and study period, with the other variables chosen based on the specific issue under investigation. It should be noted that, unless combined with matching for gonadotropins dose administered, matching for the number of oocytes cannot be expected to overcome the problem of a limited ovarian reserve. Aneuploidy raises with drug dosage, explaining why higher doses of gonadotropins can boost oocyte retrieval but not the pregnancy rates [19, 20].

A typical example of this study design, again aimed at assessing the detrimental impact of surgery on IVF outcomes, comes from Somigliana et al. (2008) [21]. They retrospectively selected women who underwent bilateral endometrioma surgery and matched them in a 1:2 ratio (*n* = 68 cases and *n* = 136 controls) by age and study period with unaffected women. Results showed that the dosage of gonadotropin was higher and the ovarian response was lower. The ORs for clinical pregnancy and live birth were 0.34 (95% CI: 0.12–0.92) and 0.23 (95% CI: 0.07–0.78), respectively [21].

### Objective of the study

**Fig. 1:**
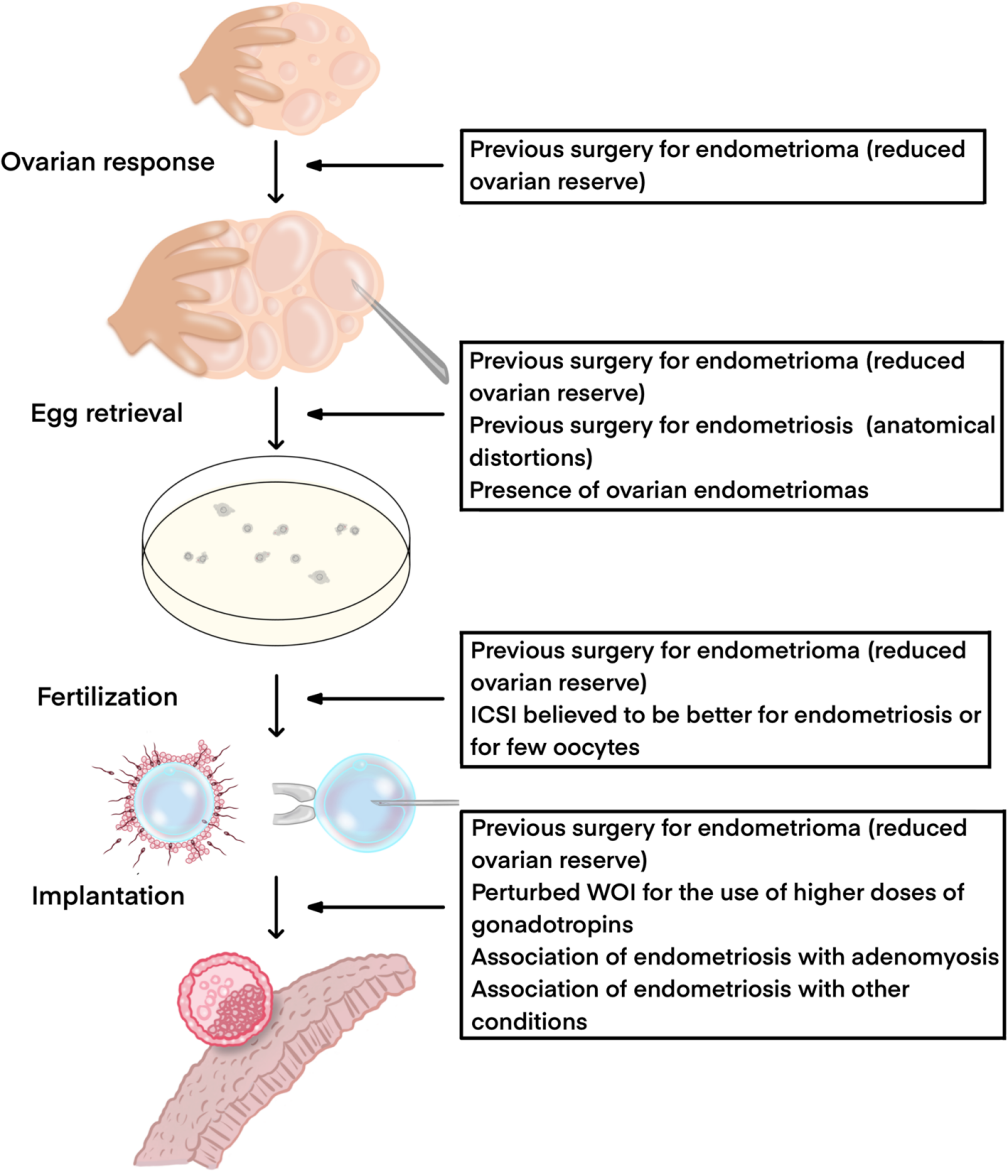
Key confounders in different steps of the IVF procedure that could influence study results on the impact of endometriosis. WOI: Window of Implantation; ICSI: Intracytoplasmic sperm injection

To better clarify the impact of endometriosis on IVF, we have herein ‘unpacked’ the steps of the process, focusing each section on studies that have tried to minimize confounders and the shortcomings of traditional observational studies. Priority was given to information from studies using the intra-patient comparison of the two ovaries or employing meticulous matching. A consistent proportion of the contributions were published by our group, reflecting a long-lasting commitment to this research area [22–26]. A list of key factors potentially interfering with the correct interpretation of assisted reproductive technologies (ART) outcomes in observational studies concerning endometriosis is illustrated in Fig. 1.

Conversely, confirmation of the detrimental effects of surgery was beyond the scope of the present study, as this topic has already been well ascertained in previous research [8–10]. Instead, the following main issues will be reviewed:

- Impact of endometriosis on ovarian response.
- Impact of endometriosis on oocyte quality.
- Impact of endometriosis on embryo implantation.

## Methods

A literature search was carried out in PubMed for the period between January 1^st^, 2000 and August 30^th^, 2023. The syntaxes used were “*endometriosis AND matched AND (art OR assisted reproductive technology OR IVF OR *in vitro* fertilization OR ICSI)*” (98 papers retrieved) and “*endometrioma AND (unilateral OR contralateral) AND (art OR assisted Reproductive technology OR IVF OR *in vitro* fertilization OR ICSI)*” (94 papers retrieved). Only studies providing reliable and unbiased information on specific steps of the IVF procedure were considered. Reviews were cited if deemed useful. No efforts were performed to identify abstracts submitted to meetings.

### Endometriosis and ovarian response to gonadotropin stimulation

According to the meta-analysis by Hamdan and co-workers, which included 17 studies for a total of *n* = 17,593 IVF cycles, a lower mean number of oocytes retrieved per cycle was demonstrated in women with endometriosis compared to controls (mean difference: − 2.0, 95% CI: − 2.9 to − 1.1) [8]. One is tempted to speculate that endometriosis per se may reduce the number of oocytes retrieved.

Notably, when assessing the endometriosis-related influence on ovarian response, some confounding factors come into play, including: (i) prior surgery, which can affect ovarian reserve and responsiveness to stimulation; (ii) the incompleteness of oocyte retrieval. Regarding this latter point, physicians are generally concerned by the risk of endometrioma infection during oocytes retrieval and tend to avoid endometrioma transfixion. Moreover, due to endometriosis, ovaries may be dislocated in the pelvis, making the retrieval more difficult (Fig. 1) [12]. Accordingly, the frequency of incomplete follicular aspiration was found to be over three times more common in affected women [27].

#### Insights from a rigorous matching design

**Fig. 2:**
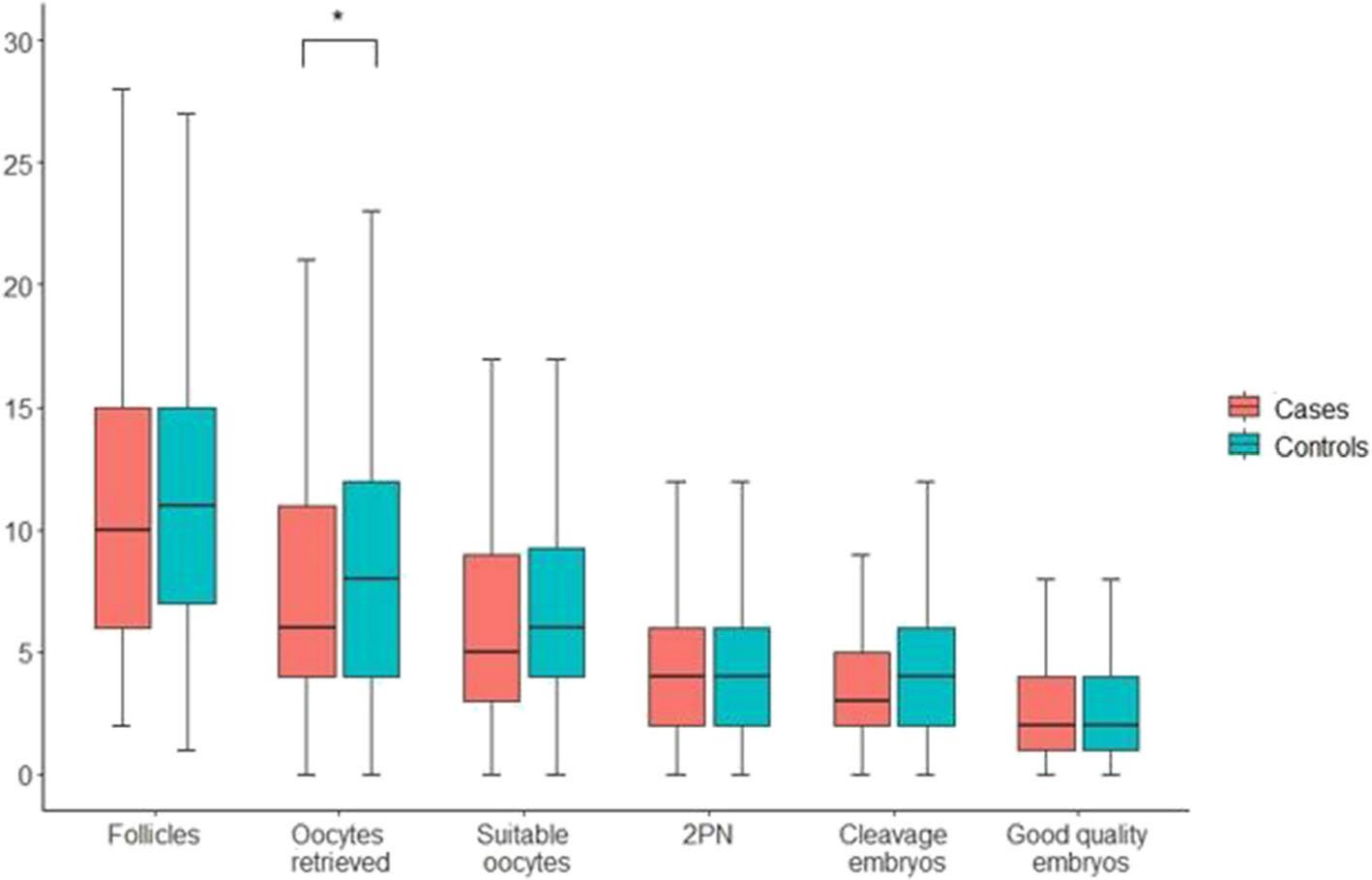
Box and whiskers plot of the number of follicles, oocytes retrieved, suitable oocytes, 2PN (fertilized oocytes), cleavage embryos and good quality embryos. Data from women with and without endometriosis are represented in red and green, respectively. A statistically significant difference emerged only for the number of oocytes retrieved (highlighted with an asterisk)

To provide an unbiased evaluation of ovarian responsiveness in women with endometriosis, we have designed a study where *n* = 248 women with endometriosis and an adequate ovarian reserve (AMH > 1.1 ng/ml) were meticulously matched to *n* = 248 controls, according to age, pharmacological regimen (same drug, same initial dose), AMH concentration and study period [23]. Prior surgery for endometriosis or the presence of ovarian endometriomas were not exclusion criteria. This study design aimed to furnish an unbiased understanding of endometriosis’s effect on ovarian response. To concomitantly assess quantitative and qualitative aspects of the ovarian response, our primary outcome was the unavailability of good quality embryos on day 3 (not pregnancy rates as this might be influenced by the concomitant presence of adenomyosis). The rate of unexpected poor response (retrieval of ≤ 3 oocytes) according to the Poseidon Group (2016) as well as the overall success rate were secondary outcomes [28]. Results obtained showed that the number of women without good quality embryos did not differ between women with and without endometriosis (16% in both groups). However, in women with endometriosis, the duration of stimulation was longer, and the number of oocytes retrieved (but not mature oocytes) was lower. The rate of unexpected poor response to ovarian stimulation differed being 13% in non-affected cases versus 23% in controls (*p* = 0.005). Notably, in subgroup analyses, such higher rate of unexpected poor responders persisted only in women who had undergone surgery for the disease. All other variables related to ovarian response showed no notable difference (results are presented in Fig. 2).

Albeit being a secondary outcome, it is worth noting that the cumulative clinical pregnancy and live birth rates were almost identical, even slightly favouring the endometriosis group (50% and 40% in endometriosis patients, and 49% and 36% in controls, respectively). Taken together, results from this study suggest that endometriosis per se does not have a major impact on folliculogenesis. The observed detrimental effect of surgery on the risk of unexpected poor response may reflect an increased difficulty in the oocyte retrieval procedure.

Another matched study published in 2017 should also be mentioned, although the sample size was smaller and the matching less scrupulous [29]. The authors retrospectively matched *n* = 119 women who had undergone surgery for endometriosis to a control group of *n* = 119 women without the disease by age, serum AMH, number of previous cycles and method of fertilization (conventional IVF or ICSI). The number of oocytes retrieved, and the number of good quality embryos were comparable. The live birth rate per cycle was also similar (27% vs 30%) [29].

#### The impact of ovarian endometriomas

The impact of endometriomas on ovarian response represents a related but independent issue. Several intra-patient comparisons between the two gonads (affected versus unaffected) have been performed to determine if unilateral ovarian endometriomas could affect ovarian response in women on ART cycles who had not previously had ovarian surgery [8, 12, 30–33]. These studies generally suggest that the presence of these cysts does not significantly impact ovarian response. Only one of these studies was prospective and reported also data on oocytes quality [34]. The number of developed follicles and oocytes retrieved were similar, being 3.7 ± 2.4 and 4.1 ± 1.7, and 4.2 ± 3.1 and 4.7 ± 2.5, respectively in the two ovaries. Fertilization and cleavage rates, and rate of high-quality embryos did not differ, being 64% and 64%, 58% and 51%, and 31% and 21%, respectively. However, the limited sample size (*n* = 29) and the small mean diameter of the endometriomas (25 ± 9 mm) hindered strong conclusions.

For bilateral endometriomas, three retrospective studies could be mentioned, of which one was very small (only *n* = 13 women) and not matched [35]. The second study, from our group, included *n* = 39 cases and *n* = 78 controls matched in a 1:2 ratio for age and study period [36]. Despite similar biomarkers of ovarian reserve, the number of follicles > 15 mm and oocytes retrieved were fewer in women with bilateral endometriomas compared to controls, being 6.2 ± 2.6 and 9.6 ± 4.8 (*p* < 0.001) and 7.1 ± 3.2 and 9.8 ± 5.5 (*p* = 0.001), respectively [36]. However, the cumulative live birth rate did not significantly differ, being 25% and 31%, respectively [36]. A third matched study enrolling *n* = 70 women with unoperated endometriomas, of whom *n* = 38 had bilateral cysts, failed to show any significant difference in serum AMH levels or number of embryos obtained. Notably, a subgroup analysis specifically focusing on these *n* = 38 women with bilateral endometriomas and their *n* = 38 matched controls was not reported [37].

A neglected but crucial aspect that could explain these inconsistencies is the size of the endometrioma. Several studies that examined the intra-patient comparison of ovarian response among women with unilateral endometriomas presented subgroup secondary analyses based on cyst diameter, suggesting a detrimental effect based on the cyst dimension [22, 38]. In general, firm conclusion could not be drawn because of the insufficient number of large endometriomas included and the nature of these analyses being secondary or exploratory. Ferrero et al. (2017) were the first to selectively focus on women with unilateral endometriomas larger than 5 cm. The intra-patient comparison showed a significant decline in ovarian response with a lower number of follicles in ovaries with endometriomas (2.6 ± 1.3) compared to healthy ovaries (4.8 ± 2.0; *p* < 0.001). Since the number of oocytes retrieved was recorded separately for the two ovaries, they were also able to report a marked difference between the affected and unaffected ovaries, which was 1.5 ± 1.1 and 3.3 ± 1.5, respectively (*p* < 0.001) [31].

A multicenter international study was then set aiming to identify the threshold of diameter above which ovarian response starts to be critically impaired [32]. The authors retrospectively included unoperated women carrying unilateral endometriomas with a mean diameter between 20 and 49 mm, and categorized them based on endometrioma size: 20–29 mm, 30–39 mm, and 40–49 mm. A negative effect on the number of developing follicles was observed only for cysts with a mean diameter from 40 to 49 mm. The median [interquartile range – IQR] number of developed follicles was 5 [3–7] and 7 [4–8] in affected and not affected ovaries, respectively (*p* = 0.01). These results suggest that a threshold of 4 cm might be used to discriminate between cysts that do and do not affect ovarian responsiveness [32].

Finally, a rather popular but poorly investigated aspect is represented by the possibility that the potential detrimental effect of endometriomas on ovarian reserve and response to gonadotropin might be progressive over time. In other words, recently developed ovarian endometriomas might initially present little to no issues whereas long-lasting lesions might pose significant risks. The biological plausibility supporting this view stems from the fact that ovarian endometriomas contain a plethora of potentially toxic agents. The long-lasting diffusion of these substances into the ovarian stroma may progressively damage and diminish the primordial follicular pool [39]. However, from the clinical point of view, this issue is controversial. Kasapoglu and coauthors repeated AMH testing at 6 months apart in *n* = 40 women with endometriomas (mean diameter 46 ± 17 mm, bilateral in 9 subjects) and *n* = 40 controls. They observed a statistically significant reduction of 26% (95% CI: 11–55%) in the formers, but no significant changes in the controls [40]. In contrast, we set up a study to retrospectively weight this aspect in women with endometriomas (average diameter of 26 ± 8 mm), who underwent more than one cycle of ovarian stimulation at intervals of more than 6 months (median 11 months, IQR 8–14 months). The contribution of the affected ovary to the overall response in terms of number of follicles retrieved remained consistent across cycles and equal to 44% (31–58%) during the first cycle and 44% (35–55%) in subsequent cycles [41]. From these two studies, we may infer that while the detrimental effects of endometriomas over time is unremarkable for small cysts, it could be significant for larger cysts.

### Endometriosis and levels of steroid hormones

According to Barnhart and coauthors, women with endometriosis have a 19% reduction of peripheral of estrogen levels at the time of ovulation trigger [7], suggesting an altered steroidogenesis. Some molecular studies support a negative influence of endometriosis on growth, steroidogenic activity, and function of granulosa cells [42]. In affected women, both granulosa cell expression of P450 aromatase (an enzyme that converts androgens to estrogen) and estrogen concentrations in the granulosa cell culture mediums were found to be reduced [43].

However, when interpreting these findings, one cannot exclude a confounding effect arising from reduced ovarian reserve, at least when addressing evidence from clinical studies. The above-mentioned study from Invernici and co-authors (2020), who carefully matched cases and controls for ovarian reserve, tends to reject the hypothesis of perturbed folliculogenesis. The serum estradiol at the time of trigger was identical, the median [IQR] being 1837 [1283–2831] and 1901 [1341–2811] pg/ml in cases and controls, respectively [23]. Reschini and co-authors (2020) designed a study specifically tailored to address this issue. Matching *n*=53 cases and *n*=53 controls by study period, age, total number of developed follicles, protocol of ovarian stimulation, type and starting dose of gonadotropin, they reported similar median [IQR] serum estrogens of 1586 [1146–2787] and 1625 [1060–2322] pg/ml, respectively [26]. Overall, available clinical evidence challenges the data from basic science studies [42]. Ovarian steroidogenesis does not seem to be affected in women with endometriosis, further supporting the idea that the disease might have minimal, if any, impact on oocyte quality.

### Endometriosis and fertilization rate

Although the number of studies included was very limited, some meta-analyses reported a reduced fertilization rate per oocyte in women with endometriosis [4, 5]. According to Horton and co-workers (2019), this finding is significant for treated patients (OR 0.92, 95% CI: 0.86–0.99, *p* = 0.03) but not for those untreated [5]. Fertilization rate seems to be more compromised in case of milder endometriosis presentations. Though, the estimation of the fertilization rate in affected cases is as well not devoid of confounding factors.

Previous studies have retrospectively compared results between ICSI and conventional IVF (c-IVF) in women with endometriosis [43]. This was based on the assumption that endometriosis itself might be responsible for a reduced oocyte competence so that ICSI, rather than c-IVF, could overcome this oocyte impairment. Comparing sibling oocytes, Komsky-Elbaz et al. have reported a higher fertilization rate when ICSI was preferred rather than c-IVF in couples with stages III–IV endometriosis [43]. However, possible biases in the analysis should be kept in mind, including: (i) maturity of oocytes is routinely established in case of ICSI and this selection bias may contribute to a higher fertilization rate per oocyte compared with unselected oocytes undergoing c-IVF; (ii) the common tendence to prefer ICSI in cases of male infertility but also to avoid total fertilization failure.

A more accurate approach for the correct assessment of this parameter would be a comparison of the fertilization rate of oocytes from women with and without endometriosis by means of the same insemination approach. Along this line, the above-mentioned study from Shebl et al. is of great interest because the authors ensured matching based on the fertilization procedure used [29]. They observed comparable fertilization rates for women requiring ICSI and a slightly lower rate among those endometriosis women treated with c-IVF (45% versus 54%, *p* = 0.03). Again, a potential bias could be introduced as the analysis was performed per oocyte (and not per woman). In a recent matched case–control study, we have demonstrated that a diagnosis of endometriosis does not negatively affect the performance of c-IVF [29]. Three-hundred and fourteen patients with endometriosis and normozoospermic partners have been matched in a 1:1 ratio with patients undergoing IVF for other indications, with respect to age (± 6 months), number of oocytes retrieved (± 1), and study period. The fertilization rates did not differ between women with and without endometriosis (median [IQR] being 78% [60–100%] and 75.0% [56–90%]; *p* = 0.24, respectively) [24]. A similar approach should be adopted for ICSI in endometriosis patients with also a male infertility factor to prove that the fertilization rate is not substantially compromised in women with endometriosis requiring ICSI. To date, it can be reasonably inferred that endometriosis does not impact the performance of c-IVF.

### Endometriosis and embryo quality and ploidy

The assessment of embryo morphology and ploidy rate in women of endometriosis, as a measure to quantify the impact of the disease on ART outcomes, is not devoid of problems. Firstly, morphological features are characterized by differences in the criteria adopted to evaluate embryo quality, leading to inconsistencies across studies. Furthermore, both embryo morphology and ploidy seem to be at some extent affected by the ovarian reserve and the dose/duration of gonadotrophin regimen used for ovarian stimulation [44]. This leads to the idea that retrospective studies addressing this question may have been confounded by the possible inclusion of affected women who have undergone surgery. Despite these possible limitations and confounders, a recent meta-analysis based on 22 studies, specifically addressing high embryo quality rate as main outcome measure, did not show any negative impact of endometriosis [45]. Women with endometriosis, including severe stages and endometriomas had similar rates of embryo formation, cleavage embryos and high-quality embryos rates compared with the control group [45].

Sanchez et al. analysed *n* = 429 ART cycles in women undergoing surgery for moderate/severe stages and compared them with *n* = 851 cycles in control patients matched for age, number of oocytes retrieved and study period [46]. No differences were reported in terms of number of cleavage stage embryos and proportion of good/fair quality embryos. In contrast, this study documented a reduced likelihood of pregnancy in the endometriosis group, which may be explained by the higher doses of gonadotropins required in the endometriosis group to achieve the same number of oocytes [19, 20]. Furthermore, the conclusions of this study are limited by the inclusion of cycles adopting only cleavage stage embryo transfer strategy, the exclusion of cycles where no embryo was obtained or all embryos were cryopreserved, and by limited attention given to the selection of controls.

Going further, Vaiarelli and coworkers have evaluated the euploid blastocyst rate per cohort of inseminated metaphase II oocytes [47]. Affected patients (*n* = 210) were matched in a 1:2 ratio to controls (*n* = 420) by IVF clinic, maternal age at retrieval, number of previous failed IVF treatments and number of metaphase II oocytes retrieved. The blastocyst rate and the embryo euploid rate per cohort of fertilized oocytes was similar between cases and matched controls, even if the blastocyst morphology was not considered.

Only two other studies have examined the euploid rate of embryos from patients with endometriosis. Results are controversial. In 2017, Juneau et al. retrospectively analysed the aneuploidy rate of 1880 blastocysts obtained from patients with endometriosis and compared them with 23,054 blastocysts from age-matched controls. They reported similar aneuploidy rates per biopsied blastocyst in the two groups [48]. In disagreement, Yan and coworkers, evaluating 7092 biopsied embryos, found a lower euploid embryo rate in women with endometriomas compared to controls (53% vs. 62%, *p* = 0.012) [49]. However, in this latter study, the statistical differences between the two groups in terms of total and starting dose of gonadotrophins used and FSH levels, question its absence of confounding factors. In this regard, based on the study design employed, results from Vaiarelli and coworkers seem the most robust [47].

### Endometriosis and embryo implantation rate

Embryo implantation potential is one of the most debated aspects of endometriosis-related infertility and IVF failure. An altered receptivity was advocated as a main reason for the lower pregnancy rate in women with endometriosis, beyond the lower ovarian reserve. A burden of literature has documented molecular and cellular alterations in the eutopic endometrium of women with endometriosis. These molecular pathways can be broadly classified into several groups including epigenetic modifiers, immune response regulators and inflammation triggers, hormonal stress inducers, epithelial-mesenchymal transition modulators [50]. Given these premises, it has been hypothesized that the communication between embryo and endometrium could be impaired, increasing the risk of implantation failure [51]. The inflammatory milieu of the pelvis has also been supposed to have some echoes in the endometrial cavity (secondary event). Regardless of the pathogenetic pathways leading to altered endometrium (i.e., whether they are primary or secondary of the disease, or both), ART is not the solution for these detrimental mechanisms. ART treatments can overcome most of the anatomic and functional impairment of the reproductive system, but they cannot heal the supposed molecular endometrial alterations.

Notably, measuring endometrial receptivity is the most challenging step in case of endometriosis. Embryo implantation is influenced by two main confounding factors. First, the low ovarian reserve reduces the rate of optimal embryos to transfer. In addition, poor responders are at higher risk of early progesterone elevation [52, 53], a condition that displace the window of implantation, therefore interfering with embryo implantation [54]. Second, endometriosis is associated with conditions that per se interfere with implantation, including adenomyosis, polyps and endometritis [3, 55–57]. Endometrial polyps and chronic endometritis are thought to exert a negative effect on endometrial receptivity [55, 57–59]. Adenomyosis is thought to prompt both uterine hyperperistalsis and fibrosis through epithelial-to-mesenchymal transition and fibroblast-to-myofibroblast transdifferentiation. Notably, the number of microvilli is reduced, steroid hormone metabolism is altered, and oxidative stress is increased in the endometrium of women with adenomyosis [3].

Clinical studies specifically designed to investigate the detrimental effect of the disease on endometrial receptivity are therefore difficult to conduct [59–63]. Analysing data derived from the ‘freeze all’ strategy could represent a way to eliminate some of the confounders. In a retrospective Chinese cohort study based on more than *n*=400 endometriosis patients undergoing frozen embryo transfer after ART treatments, affected patients were matched in a 1:3 rate with women undergoing ART due to tubal factor-related infertility, considering their age, infertility duration, serum FSH levels, antral follicular count, and BMI. Results obtained showed that endometriosis patients have lower live birth rate per transfer, as well as lower cumulative live birth rate, compared to controls [63]. However, the number of oocytes retrieved was significantly lower in affected women. As already discussed [46], this may affect the chance of pregnancy because embryos obtained with higher doses of gonadotropins or lower number of oocytes are at higher risk of aneuploidy. Accordingly, Blank et al. also observed a detrimental effect on pregnancy rates when comparing fresh transfers between women with and without endometriosis, after matching them for study period, age, parity, and embryo quality [62]. However, the number of retrieved oocytes were again significantly lower among women with endometriosis, as well as the rate of c-IVF (controls predominantly resorting to ICSI due to male factor infertility). Not surprisingly, other studies present differing views. Bishop et al. evaluated the implantation trend in three populations undergoing euploid frozen embryo transfer after ART treatments for different indications, including endometriosis, male factor, and preimplantation genetic testing for monosomic disorders. This study design overcomes the limitations of the three beforementioned studies. No difference in pregnancy outcomes, including live birth rate, were found across the groups [61]. Zimmermann et al. recently compared *n* = 195 women who had undergone surgery for stage III-IV endometriosis to a control group matched for age, BMI, serum AMH, and number of previous cycles. The observed cumulative live birth rates were 32% and 37%, respectively (*p* = 0.24) [64]. Finally, our group has recently set up a matched case–control study (*n* = 101 per group) with the aim to compare ART outcomes following single frozen embryo transfers between women with and without moderate/severe endometriosis. Remarkably, case and controls are matched not only for age, but also for number and quality of blastocysts obtained. The cumulative live birth rate per cycle did not vary between the two groups (affected: 51% *vs* healthy: 58%, *p* = 0.32) supporting a limited, if any effect of the disease on endometrial receptivity [25].

Even more interestingly, some studies have evaluated whether endometriosis would be responsible for a supposedly low implantation rate when they are recipients of donor oocytes. A retrospective study assessed the cumulative pregnancy rates in more than 10,000 oocyte donation cycles over a 10-year period. Recipients with endometriosis had similar cycle outcomes compared to other oocyte recipient groups, who received oocytes for other infertility indications, such as low ovarian response, recurrent ART treatments failure, or advanced age [65]. Overall, the concept that the uterine environment could be responsible for affecting the implantation process in women with endometriosis is challenged by the previous findings.

### Concluding remarks

While endometriosis remains an enigmatic disease from the aetiology standpoints, the mechanisms underlying its consequences on fertility and pain perception are currently better characterized. As our knowledge increases, factors that may interfere with the objective and accurate assessment of the clinical consequences of endometriosis are emerging. In this context, it is becoming evident that meta-analytic data of observational studies are not always reliable. Synthesising observational studies can lead to a high risk of within-study and across-study biases, as well as to the presence of increased heterogeneity [66].

To overcome these difficulties, we have herein reviewed available evidence on the relation between endometriosis and IVF outcomes, unpacking each step of the process, prioritizing intra-patient comparisons (that are highly informative for unilateral endometriomas) and matched studies. To note, the method of matching differed according to the specific aspect of the IVF procedure that one aimed to investigate. The main conclusions that could be disentangled from our effort are the following:

1. Endometriosis is unremarkable to ovarian response. A reduction in the response to ovarian stimulation can be detected only for endometriomas larger than 4 cm. The follicular steroidogenesis is unaffected.
2. Oocyte quality is preserved. Fertilization rate is similar, making ICSI unjustifiable. Embryological development does not differ from other forms of infertility, with no surge in aneuploidy rate.
3. Endometrial receptivity is not or minimally reduced. To note, the most informative studies supporting this perspective did not exclude women with adenomyosis, a main confounder that was expected to lower the success of the procedure. This further strengthens the idea that women with endometriosis should not be considered at increased risk of implantation failure. However, our selected evidence does not allow us to draw any conclusion on women with most advanced and disrupting forms of adenomyosis. These cases are rare, and the selected studies cannot be used to conclude that adenomyosis is unremarkable.

In conclusion, our review suggests that endometriosis does not affect IVF outcomes. Deciding different regimens of treatment or different laboratory protocols solely based on the diagnosis of endometriosis is not justified. On the other hand, it must be reminded and emphasized that the present review investigated possible sources of impairment beyond the damage to the ovarian reserve. In fact, the main relevant challenge in infertile women with endometriosis undergoing IVF is the prevention of surgically induced ovarian damage.
